# Risk stratification by anamnesis increases SARS-CoV-2 test efficiency in cancer patients

**DOI:** 10.1007/s00066-021-01853-7

**Published:** 2021-10-07

**Authors:** Christian Cornelius Arnold, Jens von der Grün, Mark Christoph Brekner, Jörg Licher, Emmanouil Fokas, Claus Rödel, Maximilian Fleischmann

**Affiliations:** 1 Department of Radiotherapy and Oncology, Goethe University, 60590 Frankfurt am Main, Germany; 2 German Cancer Research Center (DKFZ), Heidelberg and German Cancer Consortium (DKTK), Partner Site Frankfurt am Main/Mainz, Frankfurt am Main, Germany; 3 Frankfurt Cancer Institute (FCI), Goethe University, Frankfurt am Main, Germany

**Keywords:** SARS-CoV‑2 testing, COVID-19, Radiation Oncology, Radiotherapy, Real-time polymerase chain reaction (PCR) tests

## Abstract

**Purpose:**

To evaluate the impact of testing asymptomatic cancer patients, we analyzed all tests for severe acute respiratory syndrome coronavirus-2 (SARS-CoV-2) before and during radiotherapy at a tertiary cancer center throughout the second wave of the pandemic in Germany.

**Methods:**

Results of all real-time polymerase chain reaction (RT-PCR) tests for SARS-CoV‑2 performed at our radio-oncology department between 13 October 2020 and 11 March 2021 were included. Clinical data and anamnestic information at the time of testing were documented and examined for (i) the presence of COVID-19-related symptoms and (ii) virus-related anamnesis (high-risk [prior positive test or contact to a positive tested person within the last 14 days] or low-risk [inconspicuous anamnesis within the last 14 days]).

**Results:**

A total of 1056 SARS-CoV‑2 tests in 543 patients were analyzed. Of those, 1015 tests were performed in asymptomatic patients and 41 tests in patients with COVID-19-associated symptoms. Two of 940 (0.2%) tests in asymptomatic patients with low-risk anamnesis and three of 75 (4.0%) tests in asymptomatic patients with high-risk anamnesis showed a positive result. For symptomatic patients, SARS-CoV‑2 was detected in three of 36 (8.3%) low-risk and three of five (60.0%) high-risk tests.

**Conclusion:**

To the best of our knowledge, this is the first study evaluating the correlation between individual risk factors and positivity rates of SARS-CoV‑2 tests in cancer patients. The data demonstrate that clinical and anamnestic assessment is a simple and effective measure to distinctly increase SARS-CoV‑2 test efficiency. This might enable cancer centers to adjust test strategies in asymptomatic patients, especially when test resources are scarce.

**Supplementary Information:**

The online version of this article (10.1007/s00066-021-01853-7) contains supplementary material, which is available to authorized users.

## Introduction

The emergence of SARS-CoV‑2 has changed the world. Until May 2021, more than 3.4 million people around the globe died of the disease caused by the virus, called COVID-19 [1]. The infection is most harmful in the elderly population, but also in younger individuals with comorbidities. Cancer patients, particularly those under therapy, are suspected to be at higher risk for severe COVID-19 [2–6]. This has led to immense efforts in cancer centers to prevent the spread of the infection within departments [7, 8]. One strategy might be the testing of asymptomatic patients before and during anticancer treatment to detect unrecognized carriers [9]. This has been recommended in guidelines by scientific organizations such as the European Society for Medical Oncology (ESMO) [10]. Yet, evidence about the impact of systematic testing in asymptomatic cancer patients is lacking [11, 12]. For radiation oncology departments, regular screening of asymptomatic patients is resource consuming because radiotherapy regimens frequently have to be provided over a period of up to 2 months [13]. Hence, there is a clear need for efficient test strategies to manage the ongoing COVID-19 pandemic; the end of which cannot be foreseen [14, 15]. In this study, we report the results of testing asymptomatic as well as symptomatic cancer patients for SARS-CoV-2 at the Radiation Oncology Department of the University Hospital Frankfurt, Germany, during the second wave of the COVID-19 pandemic in autumn/winter 2020/2021. In addition, anamnestic information at the time of testing was assessed to evaluate possible improvements of test efficiency.

## Methods

This study was approved by the Ethics Committee of the Medical Faculty of Goethe University Frankfurt, Germany (vote number: UCT-24-2021).

Clinical data, treatment protocols and virus-related anamnesis were collected from all cancer patients receiving radiotherapy between 13 October 2020 and 11 March 2021. In addition, all SARS-CoV‑2 tests in the stated time period were evaluated. For each test, nasal and pharyngeal respiratory swabs were taken for detecting SARS-CoV‑2 RNA by real-time polymerase chain reaction (RT-PCR). All tests were performed at the Department of Virology, University Hospital Frankfurt.

Test results were documented in the clinical information system ORBIS (Agfa HealthCare) and correlated with individual clinical and anamnestic data at the time of testing, i.e., (i) presence of COVID-19 symptoms and (ii) virus-related anamnesis (see below).

In case of a positive test result, contact tracing was performed and the clinical course of COVID-19 was documented.

Furthermore, SARS-CoV‑2 infection rates in the observation period were assessed by the daily 7‑day incidences within the community of Frankfurt and nationwide.

### Testing strategy

Clinical and anamnestic data of all patients were evaluated daily. First, patients were examined via questionnaire or orally for symptoms associated with COVID-19 (i.e., fever, dyspnea, cough, anosmia, sore throat, rhinitis). Second, virus-related anamnesis was assessed and classified as high-risk if the patient had contact with a positive tested person or had been tested positive themselves within the last 14 days. In case of inconspicuous virus-related history, the anamnesis was classified as low-risk. All patients with high-risk anamnesis (irrespective of the exact risk factor) were considered as potentially infectious and consequences for the actual treatment (as described below) were similar.

A RT-PCR test was performed for every patient displaying suspicious symptoms. In case of negative result, repeat tests were conducted whenever clinically necessary.

All patients whose anamnesis was estimated as high-risk were tested, and testing was repeated after 5, 10, and in some cases 14 days.

Furthermore, all patients were tested before inpatient treatment (like chemotherapy, brachytherapy or supportive care) or outpatient chemotherapy at our day clinics. For hospitalized patients, who were scheduled for a transfer from another ward to our department, a recently conducted SARS-CoV‑2 test (< 48 h) was required. In addition, all patients on our ward were tested in a 7-day rhythm.

Because of increasing incidence in the whole population, we intensified our testing strategy in the time period concerned. From 13 November 2020 every patient was additionally tested before a treatment planning CT scan was done.

In case of a positive test result, tests to monitor the infection were performed at varying time intervals, depending on the treatment circumstances and clinical presentation of the individual.

### Safety measures within the department

During the test period, general safety measures had been implemented into the daily routine. Medical staff members and patients were required to wear surgical or FFP2 face masks at all times. Everyone was obliged to keep physical distance of at least 1.5 m, whenever possible. For that purpose, the seating arrangements in the waiting rooms were converted and time schedules were adapted to avoid an accumulation of patients. Whenever feasible, follow-up visits were postponed or carried out by telephone call only. Accompanying persons were allowed in exceptional cases only (e.g., need for translation). Prescreening by telephone and before entrance to our department via questionnaire or orally was strictly conducted every day to detect suspicious symptoms or anamnestic risk-factors, like those mentioned above.

If a patient was tested positive for SARS-CoV‑2 before treatment initiation, radiotherapy was postponed whenever feasible. If radiotherapy was considered to be urgently needed, the patient was treated under high hygiene standards per in-house protocol at the end of the daily time schedule. In patients with high-risk virus-related anamnesis and negative test result, the same protective conditions were applied.

Whenever feasible, hospitalized patients were attended to in single rooms to minimize contacts. A visitor ban on our ward was instated at the beginning of the pandemic, with an exception only for patients in the terminal stage.

## Results

From 13 October 2020 to 11 March 2021, 1056 RT-PCR tests for SARS-CoV‑2 were performed. The total number of tested patients was 543. Table 1 shows the clinicopathological characteristics of the examined cohort. The number of tests per patient ranged from 1 to 11 (median: 1). Of 543, 203 (37.4%) patients had ≥ 2 tests. Of 1056 tests, 1015 (96.1%) were carried out in patients classified as asymptomatic for COVID-19-associated symptoms and 41 of 1056 (3.9%) tests were performed in patients classified as symptomatic for COVID-19-associated symptoms.

**Table 1:** Patient characteristics

Patient characteristics	Number	Percentage
*Total*	543	100
*Gender*		
Male	291	53.6
Female	252	46.4
*Age at radiotherapy onset, median (range, years)*	65 (4–93)	–
*Karnofsky Performance Status at radiotherapy onset*		
100–90	283	52.1
80–70	171	31.5
60–50	74	13.6
40–30	15	2.8
*BMI at radiotherapy onset, median (range) [kg/m*^*2*^*], n* *=* *488*	24.7 (14.8–64.4)	**–**
< 18.5	30	6.1
18.5–30	378	77.5
≥ 30	80	16.4
*Tumor entity*		
Brain	42	7.7
Head and neck	78	14.4
Breast	80	14.7
Lung	64	11.8
Upper gastrointestinal tract	21	3.9
Lower gastrointestinal tract	35	6.4
Prostate	75	13.8
Other urological	9	1.7
Gynecological	34	6.3
Dermatological	27	5.0
Hematological	55	10.1
Sarcoma	12	2.2
Other	11	2.0
*Tumor stage*		
Localized disease	383	70.5
Metastatic disease	160	29.5
*Treatment intention of radiotherapy*		
Curative (definitive)	193	35.5
Curative (neoadjuvant/adjuvant)	181	33.3
Palliative	169	31.1
*Concomitant systemic therapy*		
None	416	76.6
Chemotherapy	122	22.5
Immunotherapy	3	0.5
Chemotherapy + immunotherapy	2	0.4
*Number of radiotherapy fractions*^a^*, median (range)*	15 (1–44)	–
*Treatment duration*^a^*, median (range, days)*	22 (1–80)	–

*BMI* Body mass index

^a^Nine patients underwent re-treatment within the observation period

In 940 of 1015 (92.6%) tests in asymptomatic patients, virus-related anamnesis was classified as low-risk. In 75 of 1015 (7.4%) tests in asymptomatic patients, virus-related anamnesis was classified as high-risk. SARS-CoV‑2 was detected in 5 of 1015 (0.5%) asymptomatic cases. Two of 940 (0.2%) tests in asymptomatic patients with low-risk anamnesis had a positive result, whereas 3 of 75 (4.0%) high-risk tests were positive. The positivity rate-ratio between asymptomatic high-risk tests and asymptomatic low-risk tests was 20 (4/0.2).

Of 41 tests, 36 (87.8%) were performed in symptomatic patients with low-risk anamnesis and 5 of 41 (12.2%) tests in symptomatic patients with high-risk anamnesis. Six of 41 (14.6%) symptomatic tests revealed a positive result. SARS-CoV‑2 was detected in 3 of 36 (8.3%) symptomatic low-risk tests and 3 of 5 (60.0%) symptomatic high-risk tests. The positivity rate ratio between symptomatic high-risk tests and symptomatic low-risk tests was 7.2 (60/8.3). Table 2 gives an overview of the test results and clinical/anamnestic information at the time of testing. Additional data on positivity rates in the patient cohort with high-risk anamnesis are listed in Supplementary Table 1.

**Table 2:** Test results and clinical/anamnestic information at the time of testing

Positive tests/Number of tests (positivity rate)			
Total: 11/1056 (1.0%)			
*Patients ***without*** COVID-19-associated symptoms* 5/1015 (0.5%)		*Patients ***with*** COVID-19-associated symptoms* 6/41 (14.6%)	
*Anamnestic low-risk*^a^ 2/940 (0.2%)	*Anamnestic high-risk*^b^ 3/75 (4.0%)	*Anamnestic low-risk*^a^ 3/36 (8.3%)	*Anamnestic high-risk*^b^ 3/5 (60.0%)

^a^i.e., no prior positive test and no contact to a positive tested person within the last 14 days before testing

^b^i.e., prior positive test or contact to a positive tested person within the last 14 days before testing

Total number of positive tested patients was 8. Three of 8 (37.5%) patients were tested positive twice. Six of 8 (75%) patients were tested positive before treatment initiation and treatment was postponed for 10–40 days (median: 24.5 days), until the patient was tested negative. Two of 8 (25%) patients were tested positive during treatment and, because of mild symptoms, radiotherapy could be continued without noteworthy interruption (0 and 1 days).

All 4 patients who were tested positive without initially having suspicious symptoms developed mild to moderate symptoms within a few days. No serious clinical course of COVID-19 was recorded. No case of infection was traced back to a source within our department. Characteristics of positive tested patients and details on clinical course as well as implications for radiotherapy are shown in Supplementary Table 2.

**Fig. 1:**
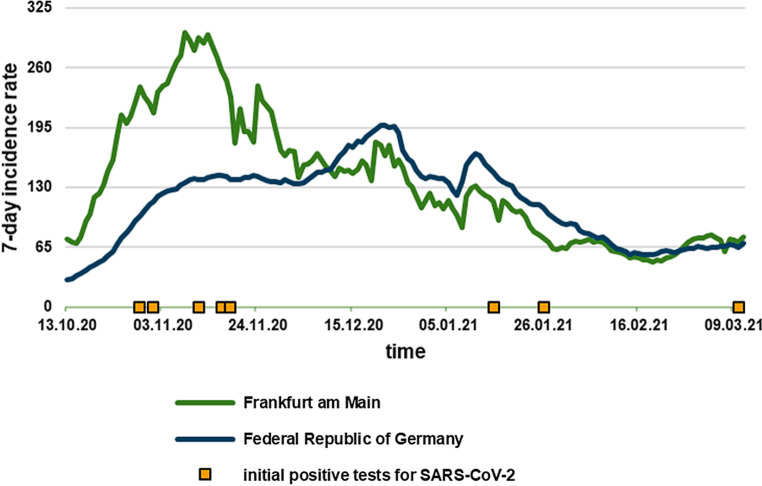
Local and national 7‑day incidence rates in the observation period and dates of patients’ initial positive test for SARS-CoV‑2. Dates on *x*-axis given as day.month.year

Local and national 7‑day incidence rates were both high within the observation period, with a higher mean 7‑day incidence rate locally (139.7 vs 112.9). Fig. 1 portrays the local and national 7‑day incidence rates over time.

## Discussion

Our study shows a relatively low prevalence of asymptomatic SARS-CoV‑2 infections in a large cohort of cancer patients before and during radiotherapy although incidence rates in the local and national population were high within the observation period. The data also reveal that efficiency of testing can be multiplied when individual risk factors of virus-related anamnesis are taken into account. The prevalence of asymptomatic carrying of SARS-CoV‑2 in cancer patients has been reported before but, to the best of our knowledge, this is the first study distinguishing positivity rates by individual anamnestic risk-factors.

Regarding previous studies, the prevalence in the cohort of cancer patients seems to be lower or at least not higher than in the whole population [16–19]. Early data from Marschner et al. were at a similar low level compared to our results and showed a positivity rate of 0.72% in asymptomatic cancer patients before initiation of radiotherapy [20]. These findings are in line with the data of Prabhu et al., who reported their systematic testing results before and during radiotherapy with SARS-CoV-2-positivity rates of 0.7% and 0.3%, respectively [21]. Modi et al. recently reported their results of preradiotherapy testing in four US institutes and also found low prevalences (0.4–2.6%) in asymptomatic patients despite a high incidence of cases in the area. The authors concluded that future test strategies may focus on risk stratification [22].

Since there is no consensus on the utility of systematic testing in cancer patients under active treatment [18–23], increasing test efficiency by risk stratification might indeed be desirable. The rational first step of risk stratification should clearly be the clinical assessment of patients. Unsurprisingly, in our study, positivity rates of patients classified as symptomatic were much higher than in the asymptomatic cohort and the utility of testing symptomatic patients is unquestionable. Nevertheless, our data reveal that risk stratification by virus-related anamnesis might increase test efficiency, also in asymptomatic patients, by a multiple. The positivity rate in asymptomatic patients with high-risk anamnesis, was much higher than in the asymptomatic low-risk cohort. Plus, only a fraction of the total test count was performed in the high-risk cohort. This highlights the usefulness of a simple daily assessment of virus-related anamnesis and suspicious symptoms and could enable care providers to select patients efficiently before testing, especially when test capacities are confined. The reverse is also true: conducting consequent, daily anamnesis can trigger rational, well-chosen tests. This might be a crucial instrument to detect asymptomatic carriers and to improve avoidance of uncontrolled viral spread.

Future test strategies for patients should not only take rigid parameters into account, but should also consider the individual treatment settings. Even though recent studies indicate that continuing radiotherapy and even systemic therapies in cancer patients with (mild) COVID-19 is feasible [24–29], testing for SARS-CoV‑2 before every systemic therapy cycle could be a useful measure to prevent treatment complications [7, 30]. Inpatient treatment significantly multiplies the transmission possibilities of asymptomatic carriers and the cohort of hospitalized patients might be particularly vulnerable for severe COVID-19. Therefore, testing before hospital admission and additionally in long-stay patients (e.g., in a 7-day rhythm) certainly have value. On the other hand, most (radio)oncological patients are treated partly or completely in an outpatient setting.

A consistent approach to detect every single (asymptomatic) SARS-CoV‑2 infection would require an enormous use of time and resources in terms of medical staff, testing material and personal equipment. In fact, most of our outpatients were only tested once, before treatment initiation. However, not a single transmission within our center was observed (the formation of an asymptomatic cluster does not seem probable). This indicates that the consistent execution of general safety/hygienic ensured feasibility and safety of anticancer treatment in our department. Hence, especially in outpatient settings, the presented risk stratification by clinical and anamnestic assessment could be of great help, allowing the test strategy to be adjusted rationally to save resources but without compromising the safety of treatments.

The overall large sample size in our study might strengthen our considerations, but definitive conclusions for future clinical practice must be drawn carefully. The most obvious limitations of this study are the retrospective design and the restriction to a single center patient cohort. To validate our classification system of low-risk and high-risk anamnesis, prospective (multicentric) trials would have to be conducted. Even though RT-PCR is established as gold standard for the detection of SARS-CoV‑2, false-negative results are a potential source of error [31]. Ongoing mutations of SARS-CoV‑2 that lead to divergent pathogenic attributes might complicate the transferability of the present data to future scenarios [32]. Moreover, the presented data have to be utilized carefully, since the results and consequences for clinical practice are highly dependent on overall incidence, societal resources and general politics.

The ongoing vaccination of the population will hopefully be the decisive game changer in everyday practice, enabling cancer centers to de-escalate various safety measures. However, for now, robust data for (long-term) vaccine efficacy for the purpose of COVID-19 prevention and reduction of virus transmittability in cancer patients is lacking [33]. As mentioned above, mutations of SARS-CoV‑2 will remain a big challenge [34]. Therefore, well-considered strategies to manage the ongoing pandemic and its subsequent waves are needed. The present study might give an indication of the direction to be taken, while noting the need for more data to ensure safe treatment settings for our patients.

## Conclusion

We evaluated the correlation between individual risk factors and positivity rates of SARS-CoV‑2 tests in cancer patients. Our data demonstrate that simple clinical and anamnestic assessment can distinctly increase SARS-CoV‑2 test efficiency. This might enable cancer centers to adjust their test strategies, especially when test capacities are confined.

## Supplementary Information

Supplementary Table 1: Positivity rates in the patient cohort with high-risk anamnesis

Supplementary Table 2: Characteristics of patients tested positive for SARS-CoV‑2
