# Supplementary material for: Risk stratification by anamnesis increases SARS-CoV-2 test efficiency in cancer patients
Source: Strahlenther Onkol. 2021 Oct 7;198(4):354–60. doi: 10.1007/s00066-021-01853-7 (PMC8494759; doi:10.1007/s00066-021-01853-7)
Supplement: Supplementary file 1 — Supplementary Table 1: Positivity rates in the patient cohort with high-risk anamnesis [file 66_2021_1853_MOESM1_ESM.docx]

**Supplementary Table 1** Positivity rates in the patient cohort with high-risk anamnesis

| Positive tests/Number of tests (positivity rate) | | | |
| --- | --- | --- | --- |
| Total: 6/80 (7.5%) | | | |
| Patients without COVID-19 associated symptoms  3/75 (4.0%) | | **Patients with COVID-19 associated symptoms**  3/5 (60.0%) | |
| prior positive test  ≤ 14 days  1/6 (16.7%) | **contact to a positive tested person ≤ 14 days**  2/69 (2.9%) | **prior positive test**  **≤ 14 days**  2/4 (50.0%) | **contact to a positive tested person ≤ 14 days**  1/1 (100%) |
