# Supplementary material for: Risk stratification by anamnesis increases SARS-CoV-2 test efficiency in cancer patients
Source: Strahlenther Onkol. 2021 Oct 7;198(4):354–60. doi: 10.1007/s00066-021-01853-7 (PMC8494759; doi:10.1007/s00066-021-01853-7)
Supplement: Supplementary file 2 — Supplementary Table 2: Characteristics of patients tested positive for SARS-CoV‑2 [file 66_2021_1853_MOESM2_ESM.docx]

**Supplementary Table 2** Characteristics of patients tested positive for SARS-CoV-2

| **Sex** | **Age** | **Tumor entity** | **Comorbidities** | **Treatment Intention** | **Concomitant Systemic Therapy** | **Number of positive tests** | **Cycle threshold values in RT-PCR** | **Reason for initial testing** | **COVID-19-related symptoms at initial testing** | **Clinical Course of SARS-CoV-2-infection** | **Delay of Treatment (days)** | **Viral Clearance (days)** |
| --- | --- | --- | --- | --- | --- | --- | --- | --- | --- | --- | --- | --- |
| f | 46 | Brain cancer | Bronchial asthma | Curative – definitive | None | 2 | 1. n.a.  2. n.a. | Suspicious symptoms | Dry cough | Few days later anosmia, symptoms persistent for at least 6 weeks | 1 | 15 |
| m | 39 | Brain cancer | Epilepsy | Curative – adjuvant | None | 1 | n.a. | Suspicious symptoms and contact to positive tested person | Fatigue, headache, diarrhoea | Total regression of symptoms after few days | 0 | - |
| f | 73 | Cervical cancer | None | Curative – adjuvant | Chemotherapy | 2 | 1. 16.4  2. 34.0 | Suspicious symptoms | Dry cough | Total regression of symptoms after few days | 27 | 27 |
| f | 35 | Brain cancer | Spastic hemiparesis, organic psycho-syndrome | Curative – adjuvant | None | 1 | n.a. | Contact to positive tested person | None | Several days later fatigue and rhinitis, total regression of symptoms after few days | 20 | 22 |
| f | 76 | Rectal cancer | Arterial hypertension, hypothyroidism, bronchial asthma | Curative – neoadjuvant | None | 1 | n.a. | Suspicious symptoms | Sore throat | Several days later dry cough, total regression of symptoms after few days | 21 | 21 |
| f | 57 | Lung cancer | Diabetes mellitus type II, bronchial asthma | Palliative | None | 1 | 26.5 | Contact to positive tested person | None | Several days later dyspnea | 12 | 40 |
| f | 52 | Tongue cancer | Hypothyroidism | Curative – adjuvant | Chemotherapy | 2 | 1. 22.7  2. 32.3 | Screening before planning CT scan | None | Several days later anosmia, total regression after few days | 14 | 10 |
| f | 73 | Cervical cancer | Arterial hypertension, diabetes mellitus type II, obesity | Curative – adjuvant | None | 1 | 18.3 | Screening before inpatient treatment | None | One day later headache, sore throat and fatigue, total regression after several days | 27 | 33 |
